# Trastuzumab deruxtecan for human epidermal growth factor receptor 2-low advanced or metastatic breast cancer: recommendations from the Japanese Breast Cancer Society Clinical Practice Guidelines

**DOI:** 10.1007/s12282-024-01550-0

**Published:** 2024-03-04

**Authors:** Masaya Hattori, Naoko Honma, Shigenori Nagai, Kazutaka Narui, Tomoko Shigechi, Yukinori Ozaki, Masayuki Yoshida, Takashi Sakatani, Eiichi Sasaki, Yuko Tanabe, Junji Tsurutani, Toshimi Takano, Shigehira Saji, Shinobu Masuda, Rie Horii, Hitoshi Tsuda, Rin Yamaguchi, Tatsuya Toyama, Chikako Yamauchi, Masakazu Toi, Yutaka Yamamoto

**Affiliations:** 1 Department of Breast Oncology, Aichi Cancer Center, Nagoya, Japan; 2 Department of Pathology, Toho University Faculty of Medicine, Tokyo, Japan; 3 Division of Breast Oncology, Saitama Cancer Center, Saitama, Japan; 4 Department of Breast and Thyroid Surgery, Yokohama City University Medical Center, Yokohama, Japan; 5 Department of Surgery and Science, Graduate School of Medical Sciences, Kyusyu University, Fukuoka, Japan; 6 Department of Breast Medical Oncology, Advanced Medical Development, The Cancer Institute Hospital of Japanese Foundation for Cancer Research, Tokyo, Japan; 7 Department of Diagnostic Pathology, National Cancer Center Hospital, Tokyo, Japan; 8 Department of Diagnostic Pathology, Nippon Medical School Hospital, Tokyo, Japan; 9 Department of Pathology and Molecular Diagnostics, Aichi Cancer Center Hospital, Nagoya, Japan; 10 Department of Medical Oncology, Toranomonn Hospital, Tokyo, Japan; 11 Advanced Cancer Translational Research Institute, Showa University, Tokyo, Japan; 12 Breast Medical Oncology Department, The Cancer Institute Hospital of Japanese Foundation for Cancer Research, Tokyo, Japan; 13 Department of Medical Oncology, School of Medicine, Fukushima Medical University, Fukushima, Japan; 14 Division of Oncologic Pathology, Department of Pathology and Microbiology, Nihon University School of Medicine, Tokyo, Japan; 15 Department of Diagnostic Pathology, Yokohama City University Medical Center, Yokohama, Japan; 16 Department of Basic Pathology, National Defense Medical College, Saitama, Japan; 17 Department of Diagnostic Pathology, Nagasaki University Hospital, Nagasaki, Japan; 18 Department of Breast Surgery, Nagoya City University, Nagoya, Japan; 19 Department of Radiation Oncology, Shiga General Hospital, Moriyama, Japan; 20 Tokyo Metropolitan Komagome Hospital, Tokyo, Japan; 21 Department of Breast and Endocrine Surgery, Kumamoto University Hospital, Kumamoto, Japan

**Keywords:** Breast cancer, Guideline, Systemic treatment, Pathological diagnosis, HER2-low

## Abstract

The Japanese Breast Cancer Society Clinical Practice Guidelines are published as timely guidance on clinical issues in breast cancer treatment in Japan. In the recent edition of these guidelines, we addressed a new clinical question 34 (CQ 34, systemic treatment part) “Is trastuzumab deruxtecan recommended for patients with unresectable or metastatic HER2-low breast cancer?” and a new future research question 7 (FRQ 7, pathological diagnosis part) “How is HER2-low breast cancer diagnosed for the indication of trastuzumab deruxtecan?”. These questions address use of trastuzumab deruxtecan in patients with unresectable or metastatic HER2-low breast cancer who have previously received chemotherapy for metastatic disease. The strengths of evidence and recommendation were determined through a quantitative and qualitative systematic review using multiple outcomes, including efficacy and safety. We conclude that trastuzumab deruxtecan is recommended for this patient population (strength of recommendation: 1; strength of evidence: moderate; CQ34) and that HER2-low expression for the indication of trastuzumab deruxtecan should be diagnosed using companion diagnostics based on appropriate criteria (FRQ7).

## Background

In March 2023, trastuzumab deruxtecan (T-DXd) was indicated in Japan for treatment of patients with unresectable or metastatic HER2-low breast cancer who have previously received chemotherapy for metastatic disease, and the Japanese Breast Cancer Society (JBCS) Clinical Practice Guidelines Committee released a new recommendation in the web revision of the JBCS Clinical Practice Guidelines 2022 edition (June 2023) [1]. This revision includes clinical question (CQ) 34: “Is trastuzumab deruxtecan recommended for patients with unresectable or metastatic HER2-low breast cancer who have previously received chemotherapy for metastatic disease?”; and future research question (FRQ) 7: “How is HER2-low breast cancer diagnosed for the indication of trastuzumab deruxtecan?”.

HER2-low is defined as HER2 immunohistochemistry (IHC)1 + or IHC2 + and in situ hybridization (ISH)-negative. HER2-low breast cancer accounts for approximately 60% of HER2-negative breast cancer cases [2, 3].T-DXd is an antibody–drug conjugate with a payload of the topoisomerase I inhibitor deruxtecan (DXd, an exatecan derivative) bound to an anti-HER2 antibody. T-DXd is recommended as a therapeutic option for HER2-positive (“IHC3 +” or “IHC2 + and ISH-positive”) metastatic breast cancer (CQ28 and FRQ12 in the JBCS guidelines [1]). Furthermore, in the recent DESTINY-Breast04 study [4], T-DXd resulted in significant improvement in progression-free survival (PFS) and overall survival (OS) in patients with unresectable or metastatic HER2-low breast cancer who had received one or two lines of chemotherapy for metastatic disease. Thus, T-DXd is approved for treatment of HER2-low unresectable or metastatic breast cancer in patients who have previously received chemotherapy for metastatic disease. However, this indication of T-DXd requires confirmation of the HER2-low status using companion diagnostics (CDx), which is covered by public health insurance in Japan.

There are urgent clinical issues that need to be addressed for treatment of HER2-low breast cancer, as a new concern in practice, and for a CDx to determine the indication for treatment. Thus, we propose this recommendation to promote treatment of HER2-low breast cancer in clinical practice in Japan with better decision-making in selection of treatment options. The recommendation process and the concepts for CQs and FRQs are consistent with those in the conventional JBCS guidelines for breast cancer [5].

### CQ34 (systemic treatment part): is trastuzumab deruxtecan recommended for patients with unresectable or metastatic HER2-low breast cancer who have previously received chemotherapy for metastatic disease?

#### Recommendation

Trastuzumab deruxtecan is recommended for patients with unresectable or metastatic HER2-low breast cancer who have previously received chemotherapy for metastatic disease. [Strength of recommendation: 1; Strength of evidence: moderate; Consensus rate: 74% (51/69)].

The DESTINY-Breast04 study is the first randomized Phase 3 study of T-DXd for patients with unresectable or metastatic HER2-low breast cancer. A literature search did not identify any other randomized phase 3 studies in this patient population. The JBCS Clinical Practice Guidelines Committee reviewed this study and determined the recommendation.

The DESTINY-Breast04 study [4] was performed as an open-label randomized phase 3 study comparing T-DXd and a treatment of physician’s choice (TPC) (capecitabine, eribulin, gemcitabine, paclitaxel, or albumin-bound paclitaxel) in 557 patients with unresectable or metastatic HER2-low breast cancer who underwent chemotherapy with 1 or 2 regimens in the metastatic setting. The median follow-up period was 18.4 months. The primary endpoint of PFS in hormone receptor-positive patients was significantly longer in the T-DXd arm than in the TPC arm [10.1 vs. 5.4 months; hazard ratio (HR): 0.51; 95%CI 0.40–0.64]. For secondary endpoints, the T-DXd arm also had significantly longer PFS in all subjects (hormone receptor-positive and -negative) (9.9 vs. 5.1 months; HR: 0.50; 95%CI 0.40–0.63), OS in hormone receptor-positive patients (23.9 vs. 17.5 months; HR: 0.64; 95%CI 0.48–0.86), and OS in all subjects (23.4 vs. 16.8 months; HR: 0.64; 95%CI 0.49–0.84).

In an exploratory analysis, hormone receptor-negative patients (i.e., triple-negative breast cancer: TNBC) in the T-DXd arm had longer PFS (8.5 vs. 2.9 months) and OS (18.2 vs. 8.3 months) than those in the TPC arm. The overall response rate (ORR) was higher in the T-DXd arm than in the TPC arm for hormone receptor-positive (52.6% vs. 16.3%) and hormone receptor-negative (50.0% vs. 16.7%) patients. The toxicity profile of T-DXd in the study was consistent with those in other studies of HER2-positive breast cancer. Interstitial lung disease (ILD) occurred in 45 patients (12.1%) in the T-DXd arm and in one patient (0.6%) in the TPC arm. Of the ILD cases in the T-DXd arm, 13 (3.5%) were Grade 1, but others were Grade 2 or higher and 3 (0.8%) were Grade 5. Grade 3 or higher adverse events occurred in 53% of patients in the T-DXd arm and 67% in the TPC arm, and included high incidences (T-DXd vs. TPC) of neutropenia (13.7% vs. 40%), anemia (8.1% vs. 4.7%), fatigue (7.5% vs. 4.7%), and nausea (4.6% vs. 0%).

#### Decision on recommendation

The evidence for CQ34 was determined to be “moderate” because only one well-planned high-quality randomized controlled trial was available. Although adverse events, including nausea and ILD, were higher with T-DXd, there were also significant improvements in OS (the most important endpoint), PFS, and ORR. There was a similar trend for improved prognosis in TNBC cases. Based on these results, the benefits of T-DXd were found to outweigh the risks. After two rounds of voting, a rating of “Strongly recommended” was determined.

### FRQ7 (pathological diagnosis part): how is HER2-low breast cancer diagnosed for the indication of trastuzumab deruxtecan?

#### Statement

Diagnosis is required using a CDx covered by health insurance. Ventana ultraView Pathway HER2 (4B5) was used as a CDx to assess low HER2 expression in the DESTINY-Breast04 study [4]. The Food and Drug Administration (FDA) and the Japanese Ministry of Health, Labour and Welfare approved 4B5 (Table 1; global recommendation protocol) as a CDx for assessment of the indication for T-DXd in patients with unresectable or metastatic HER2-low breast cancer who have previously received chemotherapy for metastatic disease. To determine the indication of T-DXd for metastatic HER2-low breast cancer, immunostaining using a CDx and scoring of the results using guidelines for the ASCO/CAP HER2 test [6] and CDx decision guide are recommended (https://www.accessdata.fda.gov/cdrh_docs/pdf/P990081S047D.pdf). The specimens include biopsy or surgery samples from primary or metastatic lesions. Pre-analytical factors including fixation conditions may have an effect on assessment of low HER2 expression [6, 7].

**Table 1:**
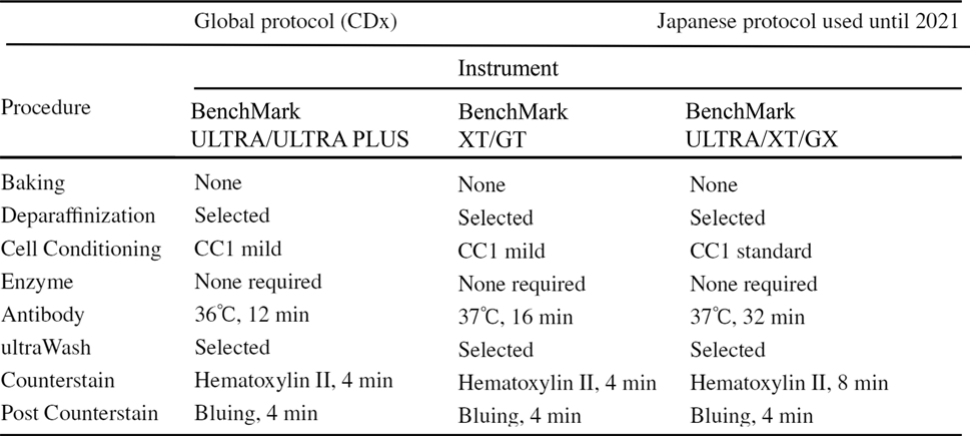
Comparison of staining procedures for Ventana ultraView PATHWAY HER2 (4B5) between the global protocol (CDx) and the Japanese protocol used until 2021 (provided by Roche Diagnostics K.K.)

#### Reassessment of low HER2 expression

Since health insurance approval (May 1st, 2023), administration of T-DXd in patients with unresectable or metastatic HER2-low breast cancer in Japan requires prior assessment of low HER2 expression using a CDx. Before this date, use of T-DXd based on an assessment of low HER2 expression (including assessment by 4B5) was not covered by public health insurance in Japan, and reassessment using a CDx is necessary. Patients who should undergo reassessment are those who were found to be negative (“IHC0”, “IHC1 +” or “IHC2 + and ISH-negative”) in previous HER2 tests. The same sample may be reassessed as different from the previous results. The causes include (1) differences in staining due to reagents and staining conditions, (2) discrepancies among diagnosticians, and (3) tumor heterogeneity: (1) CDx (4B5 based on the global recommendation protocol) has a tendency to give a lower level compared to other in vitro diagnostics [8] and 4B5 used until 2021 in accordance with the Japanese protocol (Table 2); (2) diagnosis of IHC0 and 1 + , a cut-off for low HER2 expression, depends on fine staining, and in addition, IHC0 and 1 + are both categorized as HER2-negative, with inconsistencies among pathologists [9, 10]; and (3) HER2 expression may be heterogeneous in the same tumor depending on the primary lesion site, time-dependent changes including therapeutic effects, and various primary and metastatic lesions that may give different values for low HER2 expression [8, 11]. For patients with HER2-positive tumors (“IHC3 +”, “IHC 2 + and ISH-positive” or “ISH-positive”), reassessment of HER2 is not necessary because T-DXd is already indicated for HER2-positive tumors diagnosed with conventional in vitro diagnostic agents.

**Table 2:** Comparison of immunohistochemical results for Ventana ultraView PATHWAY HER2 (4B5) between the global protocol (CDx) and the Japanese protocol used until 2021 (provided by Roche Diagnostics K.K.)

	Japanese protocol used until 2021				
	3 +	2 +	1 +	0	Total
Global protocol (CDx)					
3 +	24	0	0	0	24
2 +	0	8	0	0	8
1 +	0	1	7	0	8
0	0	0	3	26	29
Total	24	9	10	26	69

## Conclusions

With expansion of the indication of T-DXd for HER2-low breast cancer in Japan, a new CQ and a new FRQ on T-DXd were included in the 2022 web revision of the JBCS Clinical Practice Guidelines [1]. T-DXd is recommended for patients with unresectable or metastatic HER2-low breast cancer who have received one or two prior chemotherapies for metastatic disease (Strength of recommendation: 1; Strength of evidence: moderate, CQ34, systemic treatment part). HER2-low expression as an indication for T-DXd should be diagnosed using a CDx based on appropriate criteria (FRQ7, pathological diagnosis part).

## Data Availability

This manuscript is not an original article and dose not contain any original data.
